# Medicinal Plants as a Drug Alternative Source for the Antigout Therapy in Morocco

**DOI:** 10.1155/2020/8637583

**Published:** 2020-11-23

**Authors:** Nour Elhouda Daoudi, Mohamed Bouhrim, Hayat Ouassou, Mohamed Bnouham

**Affiliations:** Laboratory of Bioresources Biotechnology Ethnopharmacology and Health, Department of Biology, Faculty of Sciences, University Mohamed I, Oujda, Morocco

## Abstract

**Background:**

The gout is a metabolic disease that is associated with a high level of uric acid in the blood. This disease is treated with some medications that aim to reduce serum urate levels. However, the use of various medicines leads to the appearance of some side effects, hence the importance of using other treatments based on natural resources.

**Objective:**

This study presents some medical treatments, their side effects, and some plants that are used for gout management in Morocco in the aim to valorize them.

**Methods:**

We have been consulting various English publications in PubMed, Web of Science, and ScienceDirect published between 1991 and 2019 using the following keywords “drugs,” “gout,” “Morocco,” “medicinal plants,” “*in vitro*,” and “*in vivo*” terms. Then, we have classified the medicines, according to their action mechanisms, and we have cited some species that were reported in Moroccan pharmacopeia as antigout.

**Results:**

Three methods of the gout medical management were cited in this work: xanthine oxidase inhibitors, uric acid excretion enhancer, and uricase recombinant. However, it was found that these treatments had various side effects. We have described 23 species, and some of them showed experimentally an antigout effect by blocking the “xanthine oxidase” enzyme. These plants belong to 11 families. Lamiaceae represents the most dominant family with six species followed by Asteraceae with two species. Colchicine isolated from *Colchicum autumnale* is the most known compound for its efficiency towards gout.

**Conclusion:**

This work summarized different treatments particularly medicinal plants that are used in Morocco to treat gout disease by blocking uric acid secretion. However, several studies are needed to valorize these antigout natural sources.

## 1. Introduction

Gout is a chronic disorder, which is known also as “a disease of kings.” Sir Alfred Baring Garrod discovered it in 1848 [1]. This disease is characterized by the deposition of urate crystal in several tissues [2]. It affects many joints, particularly the first metatarsophalangeal joint of the foot. Additionally, it attacks the arms, knees, and ankles [3]. Mostly, this disease is associated with many risk factors as hyperuricemia, genetic factors, metabolism syndrome, dietary factors, alcohol consumption, osteoarthritis, kidney failure, diuretic, and some medication uses [4].

The most frequent inflammatory arthritis in the world is gout. It is characterized by prevalence higher than 1% in the occidental countries, especially in Europe and North America [1]. Actually, recent works suggest that men have a higher prevalence and incidence of gout than women [1, 4]. Indeed, it is necessary to take this disease seriously because it is associated with various health complications as cardiovascular risk, hypertension, chronic kidney disorder, obesity, and metabolic syndrome [5–7].

**Figure 1:**
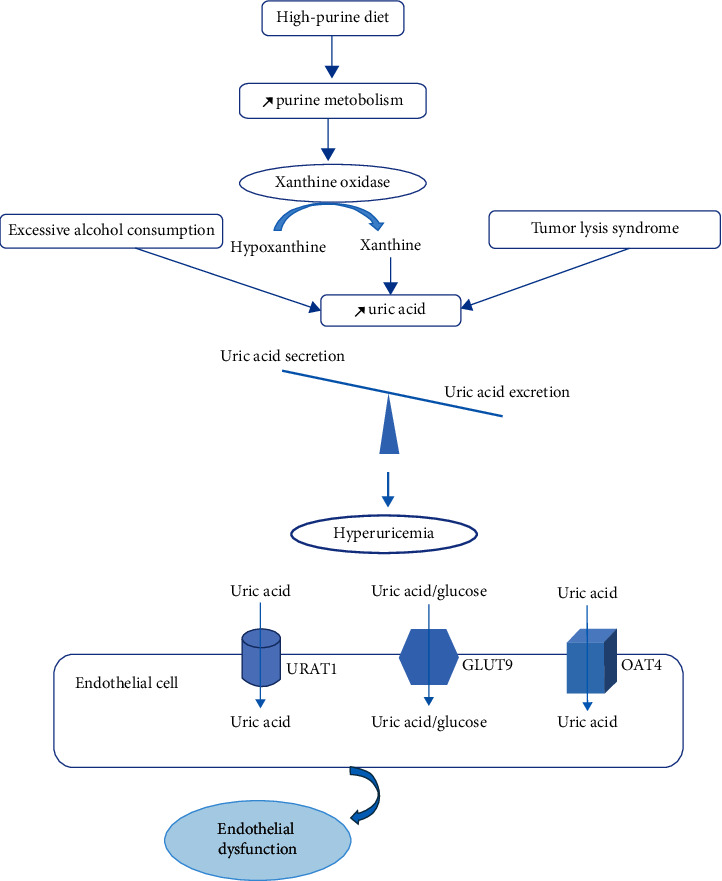
Mechanisms underlying hyperuricemia.

The main cause of hyperuricemia is the unbalance between the production and excretion rate of uric acid. Purines principally degraded by a hepatic enzyme named xanthine oxidase are involved in the appearance of hyperuricemia. A high-purine diet that leads to purine metabolism raise, excessive alcohol consumption, and tumor lysis syndrome associated with the alteration of a large number of cells, contributing to the increased uric acid production. Indeed, a reduction in excretion and rising reabsorption of uric acid causes hyperuricemia. Furthermore, uric acid transporters are required in uric acid renal handling which consists of tubular reabsorption/secretion and glomerular filtration. In fact, reabsorption-related proteins include generally glucose transporter 9 (GLUT9), organic anion transporter 4 (OAT4), and urate anion transporter 1 (URAT1). However, secretion-related transporters include OAT1, OAT3, multidrug resistance protein 4 (MRP4/ABCC4), and breast cancer resistance protein (BCRP/ABCG2) [8] (Figure 1).

Generally, gout is treated with some medications to lower the level of the serum urate by the inhibition of the xanthine oxidase in the liver. Among those treatments, we have febuxostat and allopurinol [9]. Colchicine extracted by *Colchicum autumnale* is another kind of gout therapy [10]. Unfortunately, these drugs have various side effects. For example, allopurinol causes nephrolithiasis, renal toxicity, liver necrosis, and allergic reactions [11]. For these, people seek better alternatives based on the use of medicinal plants because of their availability, easy accessibility, and people believe that natural products are not harmful [12]. Morocco is characterized by flora diversity, and it contains 4200 species in which 800 are considered therapeutic herbs [13]. The medicinal plants of this country have been used traditionally to treat several complications as gout. In fact, several plants were reported in pharmacopeia as antigout products, and most of them have shown experimentally this activity. Although, these plants remain underexploited and require other studies in order to confirm this effect and to identify different molecules responsible for this beneficial effect. Therefore, in this review, we have summarized information about medical treatment and plants that are used for gout management in Morocco with the aim to valorize the Moroccan bioresources. For this reason, we have been consulting various English publications in PubMed, Web of Science, and ScienceDirect databases, published between 1991 and 2019.

## 2. Medical Treatment

The principle of gout management is based on reducing and maintaining the serum urate at the normal value (<6.0 mg/d) [9]. Generally, there are three methods employed to reach this objective. The first one allows inhibiting the xanthine oxidase enzyme (allopurinol and febuxostat) and then blocking the production of uric acid and their precursor. The second one is intended to enhance uric acid excretion. The third strategy is based on the administration of recombinant uricases to convert uric acid to allantoin (Table 1) [9, 20].

## 3. Plants Used Traditionally to Treat Gout in Morocco

Medicinal plants have a prominent place in Moroccan phytotherapy as they contain several bioconstituents and antioxidants that are responsible for their beneficial effects. These plants and their compounds can be used as alternative or complementary medicines against gout pathology.  Table 2 classifies medicinal plants that are used traditionally in Morocco to manage gout disease.

**Figure 2:**
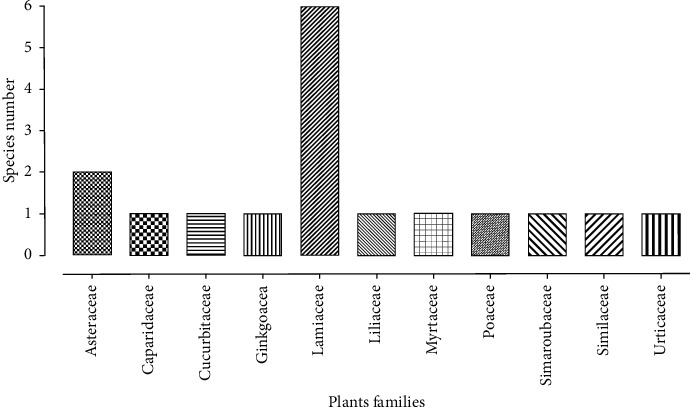
Repartition of the different antigout plant families.

Figure 2 shows the different families of the plants that had an antigout effect. In fact, the Lamiaceae family is the most dominant with six species, followed by Asteraceae with two herbs and the other families, which contain only one species for each one.

## 4. Discussion

Gout is a metabolic disease, characterized by burning, acute arthritis, and pain, which are the consequences of monosodium urate deposition into the joints [49, 50].

The release of interleukin-1 *β* (IL-1 *β*), which is a cytokine, plays a key role in the initiation of the gout disease. Actually, the crystals lead to the activation of monocyte and then releasing caspase-1, which induces the IL-1 *β* secretion [51]. The blocking of this cytokine represents an effective treatment of acute and chronic gout. Among IL-1 *β* inhibitors that are used, there are anakinra, canakinumab, rilonacept, and pralnacasan [52–55].

Diet plays also a role in the decrease of the uric acid level. In fact, beer, red meat, and sugar-sweetened beverages are responsible for the appearance of gout [20]. In the other hand, it was reported that some food consumption such as “cheery juice” (*Prunus cerasus*; Rosaceae), food that contains a high amount of vitamin C; coffee; and dairy products could maintain the uric acid's rate at normal values and then the management of gout [56, 57].

Experimentally, there are several animal models of gout which are mainly monosodium urate crystal deposition-induced acute inflammation, potassium oxonate-induced hyperuricemia, and carrageenan-induced inflammation. In fact, uric acid is considered as a weak organic acid, and it is able to be ionized to monosodium urate crystal at pH = 7.4 and at 37°C. Consequently, uric acid and urate crystals could deposit into the tissues, particularly joints and kidney, contributing to tissue damage [8]. The formation of monosodium urate crystals lead to the activation of macrophages that play a role in the secretion of inflammatory cytokines as IL-1 *β*. These mediators initiate with complement of (activated at crystals surfaces) a neutrophilic influx that is pathophysiologic feature of acute-gout. During the infiltration, crystals activate neutrophils, which contribute to the production of additional proinflammatory mediators such as arachidonic acid products (prostaglandine E2 and leukotriene B4). Monosodium urate crystals could persist into the joint fluid between attacks, which suggest that the inflammatory potential of monosodium urate crystals may be modulated by synovial fluid elements [58]. Potassium oxonate is a selective competitive inhibitor of uricase that play a key role in the conversion of uric acid to allantoin. Then, it induced hyperuricemia in rodents by blocking the effect of liver uricases [59]. Indeed, it was reported that potassium oxanate-induced hyperuricemic in rats developed a high level of renal proteins (URAT1 and GLUT9) and decreased the level of OTA1. However, the involved mechanisms are not clear yet [60]. The carrageenan-induced paw edema is another experiment model of gout that involves several inflammatory mediators such as TNF-*α* and IL-1 *β*. In fact, TNF-*α* triggers IL-1 *β* release and cytokine-induced neutrophil chemoattractant 1 which play a key role in the stimulation of the prostaglandins synthesis by cyclooxygenase-2. Furthermore, TNF-*α* activates iNOS responsible for NO synthesis, and then the responses of neutrophils increases to inflammatory stimuli [61].

Several agents are implicated in the treatment of gout disease as cyclooxygenase inhibitors, steroids, anti-inflammatory drugs, xanthine oxidase inhibitors, uricosuric, and uricases agents. Although these drugs are efficient, they are associated with various side effects such as digestives disorders, hepatotoxicity, renal dysfunction, hypersensitivity reaction, and skin allergy [62]. Actually, people search for alternatives that are characterized by their availability, fewer undesirable effects, and lower costs. These alternatives are based on medicinal plants.

In this review, we are citing some natural sources that are used traditionally in Morocco to treat gout disease. The table classifies 23 species, their scientific names, families, the traditionally used parts, traditional use method, experimental used part, extraction methods, and the experimental study towards gout, and we are reporting the toxic plants. In fact, the cited plants had shown experimentally an antigout effect by blocking and inhibiting “xanthine oxidase” which is the enzyme that catalyzes xanthine and hypoxanthine to uric acid. Xanthine oxidase inhibitors play an essential role to reduce uric acid levels, oxidative stress, and inflammation in the kidney and are able to prevent glomerular hypertension, afferent arteriolar, and ischemic renal histologic changes [63]. Besides, plants contain bioactive compounds that can enter into the active site of xanthine oxidase, producing a complex xanthine oxidase-bioactive compound through hydrophobic forces, related with surrounding xanthine oxidase amino acid and occupied the active site, thus preventing the entrance of substrate and inhibiting uric acid synthesis [64]. The histogram presented in this review shows the different families to which the studied plants belong. The Lamiaceae family is the most dominant family with six species. Recently, it was reported that the phenolic compounds isolated from the Lamiaceae herbs had anti-inflammatory and antioxidant actions [65, 66]. Besides, species belonging to this family had shown a potent xanthine oxidase inhibition, and it was recommended to use them to prevent and treat gout disease [67].

*Capparis spinosa L.* is the plant that has shown the highest inhibitory effect on xanthine oxidase activity than the other plants. The chloroform and ethyl acetate aerial part extract have shown a lower value of IC_50_ (0.023 and 0.09 mg/mL, respectively), followed by chloroform extract of root (0.32 mg/mL), which means that these extracts are characterized by a potential activity on xanthine oxidase effect [27]. However, *in vitro* assay corresponds to *in vitro* enzymatic reactions of the enzyme xanthine oxidase. It allows to investigate directly the effect of plants on the xanthine oxidase inhibition activity. These studies do not require the use of a high number of animals in tests. However, it does not take into account the bioavailability of the principles active agents. Therefore, in vitro analysis is not enough and must confirmed by *in vivo* assays; using the different experimental animal models of gout.

Several phytochemical studies have reported the presence of alkaloids, terpenoids, saponins, phenolic compounds, carotenoids, and tocopherols in *C. spinosa* L. [68] that may act as xanthine oxidase inhibitors [64]. Fruits and leaves parts of this plant have shown the presence of phenolic and flavonoid compounds, particularly caffeic acid, catechin, chlorogenic acid, coumarin, ferulic acid, kaempferol, luteolin, quercetin, resveratrol, rutin, syringic acid, and vanillic acid which possesses an important xanthine oxidase inhibition [64, 69–72]. Furthermore, the roots are characterized by the presence of capparispine, cadabicine 26-O-*β*-D-glucoside, capparispine 26-O-*β*-D-glucoside, stachydrine, and 3-hydroxy-7-methoxy-2-methyl-4H-1,4-benzoxazine-4-carbaldehyde [73].

Colchicine is a natural drug that is isolated from *Colchicum autumnale* [10]. For almost 250 years, it was used as an anti-inflammatory agent [74]. Now, this alkaloid is used to manage gout disease, neurologic disability, familial Mediterranean fever, liver cirrhosis, amyloidosis, scleroderma, and Behcet's disease [75]. However, it can be toxic as it may cause gastrointestinal disorder and respiratory paralysis. For this reason, it is recommended to administer it according to therapeutic guidelines [76].

Gout has an important relationship with diabetes. Actually, the prevalence of gout in type 1 and 2 diabetes was, respectively, 1.9% and 10.12% [77]. Besides, many studies showed that people suffering from gout have a higher risk to develop type 2 diabetes [78]. Hyperuricaemia leads kidney failure, which is the consequence of crystal deposition in the renal tract. Moreover, type 2 diabetes can further aggravate this consequence [79].

Patients that had both gout and diabetes mellitus are more probable to have a higher mean of triglycerides, high-density lipoprotein cholesterol, renal dysfunction, lower hemoglobin, and peripheral-neuropathy. Besides, it was reported that these patients are more likely to be heavy, older, and men [77].

## 5. Conclusions

The present work showed that gout is treated with different methods used to block and inhibit uric acid secretion. The first strategy adopted by Moroccan people is based on medication uses which are associated with several adverse effects. However, the second method is more safe as it focused on the utilization of plants and their constituents that take an important place in Moroccan phytotherapy. In fact, this review classifies 23 species that have used traditionally in Morocco to treat gout and have shown experimentally significant xanthine oxidase inhibition: *Ailanthus altissima, Artemisia herba-alba, Capparis spinosa* L*., Caryophyllus aromaticus* L*., Citrullus colocynthis* L*., Colchicum autumnale* L*., Ginkgo biloba* L*., Hyssopus officinalis* L*., Lavandula angustifolia, Melissa officinalis* L*., Mentha spicata* L., *Rosmarinus officinalis* L*., Smilax officinalis., Smilax medica Schltdl. and Cham., Smilax syphilitica, Smilax aristolochiifolia Mill., Smilax febrifuga Kunth., Smilax regelii Killip and C.V. Morton, Smilax aspera L., Solidago virgaurea* L*., Thymus vulgaris* L*., Urtica dioica* L., and *Zea mays* L. However, several studies are needed to valorize these plants in order to use them to manage and maintain gout disease.

## Figures and Tables

**Table 1:** The gout treatment drugs and their side effects.

Gout treatment options	Drugs	Effect	Doses (mg/day)	Side effects
Xanthine oxidase inhibitors	Allopurinol	Reduces the level of blood uric acid [9]	50–800 [9]	(i) Digestive disorders
				(ii) Hypersensitivity syndrome
				(iii) Hepatotoxicity
				(iv) Skin reactions [14]
	Febuxostat	Reduces the level of blood uric acid [9]	80–120 [9]	(i) Digestive disorders
				(ii) Headache
				(iii) Liver problems
				(iv) Rash in the skin [9]

Uricosuric agents	Probenecid	Inhibition of the uric acid reabsorption [15]	—	(i) Gastrointestinal tract irritation
				(ii) Anorexia
				(iii) Skin reactions [9]
	Benzbromarone		100	(i) Hepatotoxicity [16]
	Lesinurad	Inhibition of the uric acid reabsorption [17]		(i) Gastroesophageal reflux disease
				(ii) Headache
				(iii) Influenza associated with kidney failure [9]
	Losartan/fenofibrate	Lowering urate formation [18]		

Uricases	Rasburicase	The recombinant form of uricases		(i) Infusion problems [9]
				(ii) Antibody formation [19]
				(iii) Anaphylaxis [9]

**Table 2:** List of plants used in Moroccan alternative medicine as antigout.

Scientific name	Family	Traditional used part	Traditional method of use	Experimentalused part	Extraction methods	Experimental study	Results	Toxicity	Ref.
*Ailanthus altissima*	Simaroubaceae	Flowers	Infusion	Flowers	Decoction (distilled water)	*In vitro* (0.1, 0.5, 1.0, and 2.0 mg/mL)	Inhibition of xanthine oxidase	No	[21–23]
*Artemisia herba-alba*	Asteraceae	Flowers	Infusion	Flowers	Decoction (distilled water)	*In vitro* (0.1, 0.5, 1.0, and 2.0 mg/mL)	Inhibition of xanthine oxidase	No	[21, 24, 25]
*Capparis spinosa L.*	Caparidaceae	Fruit	Mixed the dried fruit with honey	Root and aerial part	Maceration (distilled water, methanol, hexane, chloroform, and ethyl acetate)	*In vitro*	Inhibition of xanthine oxidase (crude extract of root, IC_50,_ 0.32, 0.36, and 4.32 mg/mL) (crude extract of aerial part, IC_50,_ 0.023, 0.09, and 1.35 mg/mL)	No	[26–28]
*Caryophyllus aromaticus L.*	Myrtaceae	Flowers	Infusion	Flowers	Decoction (distilled water)	*In vitro* (0.1, 0.5, 1.0, and 2.0 mg/mL)	Inhibition of xanthine oxidase	No	[21, 24, 29]
*Citrullus colocynthis L.*	Cucurbitaceae	Pulp	Decoction (water and honey) and maceration	Leaves	Soxhlet extraction (hexane, chloroform, and methanol)	*In vitro*	Inhibition of xanthine oxidase IC_50_ = 0.58 IC_50_ = 1.8 IC_50_ = 5.96	Toxic LD_50_ = 2500 mg/kg	[26, 30, 31]



*Colchicum autumnale L.*	Liliaceae	Seeds and capsule	—	—	—	—	Antigout action	Toxic	[10, 31]
*Ginkgo biloba L.*	Ginkgoaceae	Leaves	Infusion	Leaves	Decoction	*In vitro* (0.1, 0.5, 1.0, and 2.0 mg/mL)	Inhibition of xanthine oxidase	No	[21, 32–34]
*Hyssopus officinalis L.*	Lamiaceae	Flowers	Infusion	Flowers	Decoction	*In vitro* (0.1, 0.5, 1.0, and 2.0 mg/mL)	Inhibition of xanthine oxidase	No	[21, 35–37]
*Lavandula angustifolia*	Lamiaceae	Flowers	Infusion	Flowers	Decoction	*In vitro* (0.1, 0.5, 1.0, and 2.0 mg/mL)	Inhibition of xanthine oxidase	No	[21, 26, 37]
*Melissa officinalis L.*	Lamiaceae	Leaves	Infusion	Leaves	Decoction	*In vitro* (0.1, 0.5, 1.0, and 2.0 mg/mL)	Inhibition of xanthine oxidase	Hepatotoxic	[21, 26, 38]
*Mentha spicata L.*	Lamiaceae	Leaves	Infusion	Leaves	Decoction	*In vitro* (0.1, 0.5, 1.0, and 2.0 mg/mL)	Inhibition of xanthine oxidase	No	[21, 39, 40]
*Rosmarinus officinalis L.*	Lamiaceae	Leaves	Infusion	Leaves	Decoction	*In vitro* (0.1, 0.5, 1.0, and 2.0 mg/mL)	Inhibition of xanthine oxidase	No	[21, 24, 37]
*Smilax officinalis* *Smilax medica* *Smilax syphilitica* *Smilax aristolochiifolia Mill* *Smilax febrifuga Kunth.* *Smilax regelii Killip* and *C.V. Morton Smilax aspera L.*	Similaceae	Root	Cooking the herb with the meat.	Rhizome	Maceration	*In vitro*	Inhibition of xanthine oxidase IC_50_ = 33% (methanol extract) IC_50_ = 96% (water extract)	No	[26, 41, 42]
*Solidago virgaurea L.*	Asteraceae	Leaves	Infusion	Leaves	Decoction	*In vitro* (0.1, 0.5, 1.0, and 2.0 mg/mL)	Inhibition of xanthine oxidase	No	[21, 43, 44]
*Thymus vulgaris L*	Lamiaceae	Leaves	Infusion	Leaves	Decoction	*In vitro* (0.1, 0.5, 1.0, and 2.0 mg/mL)	Inhibition of xanthine oxidase	No	[21, 45, 46]
*Urtica dioica L.*	Urticaceae	Leaves	Infusion	Leaves	Decoction	*In vitro* (0.1, 0.5, 1.0, and 2.0 mg/mL)	Inhibition of xanthine oxidase	No	[21, 24, 47]
*Zea mays L.*	Poaceae	Stigma	Infusion	Stigma	Decoction	*In vitro* (0.1, 0.5, 1.0, and 2.0 mg/mL)	Inhibition of xanthine oxidase	No	[21, 45, 48]

## Data Availability

No data were used to support this study.
